# DNA sequencing in oncology: a focus group study on a duty to recontact

**DOI:** 10.1080/20565623.2024.2432233

**Published:** 2024-11-22

**Authors:** Noor A.A. Giesbertz, Lars S. Assen, Wim H. van Harten, Annelien L. Bredenoord

**Affiliations:** a Department of Genetics, The Netherlands Cancer Institute, Amsterdam, The Netherlands; b Department of IQ Health, Radboud University Medical Center, Nijmegen, The Netherlands; c Department of Psychosocial Research and Epidemiology, The Netherlands Cancer Institute, Amsterdam, The Netherlands; d Health Technology and Services Research, University of Twente, Enschede, The Netherlands; e Erasmus School of Philosophy, Erasmus University, Rotterdam, The Netherlands

**Keywords:** Oncology, ethics, genetics, recontact, focus groups, DNA sequencing, qualitative study, cancer care

## Abstract

**Introduction:**

Particularly in genetics, former results can gain new meaning in the course of time. This raises questions about when professionals should recontact patients with new information. The aim of this focus group study is to clarify how different stakeholders in oncology think about the extent and limits of a duty to recontact.

**Materials and methods:**

One focus group with oncology patients (n = 12) and two groups with healthcare professionals (total n = 13) were conducted. In general, there was support for recontacting patients. The scope and extent of this duty was, however, perceived differently. Differences and similarities on the following six contextual factors are discussed: information features, costs and efforts, personal preferences, who is contacted, clinic or research setting, and time.

**Discussion:**

Oncology patients were clear in their wish to receive updates while the professionals were more hesitant to consider recontact as a standard of care. This is not surprising as recontacting patients with new information would mean a shift from a *patient-initiated* approach toward an *information-initiated* approach. This entails a different way of offering healthcare. Furthermore, the question is not only what professionals’ responsibilities are, but how to design a system that complies with patients’ wishes to receive updates.

## PLAIN LANGUAGE SUMMARY

In genetics, new information can sometimes change the interpretation of past results. This raises the question of when doctors should reach out to patients with updated information. A study was done to understand how different people involved in cancer care feel about this responsibility. The study included one group of cancer patients (12 people) and two groups of healthcare professionals (13 people total).

Overall, there was support for the idea of recontacting patients with new information, but opinions varied on how and when this should happen. The study looked at six key factors: the type of information, costs and efforts involved, personal preferences, who should be contacted, whether the setting is a clinic or a research environment, and the timing.

Patients generally want to be updated with new information, but healthcare professionals were more cautious about making this a standard practice. This hesitancy is understandable because it would change the way care is provided—from patients asking for updates to doctors proactively providing them. The study suggests that it’s important not only to define the responsibilities of healthcare professionals but also to figure out how to create a system that meets patients’ desires to be kept informed.

## 1. Introduction

In past decades, the developments of high-throughput technologies have enabled the sequencing of a person’s entire exome or genome [1]. As a result, our knowledge and understanding of genetics and genomics has vastly increased. DNA-diagnostics have become a cornerstone in various medical fields [2]. Also, in oncology, genomic approaches are developed and have the potential to advance the prevention, screening and treatment of cancer [3–5]. These rapid developments provide new knowledge and possibilities, while simultaneously raising ethical questions. One central ethical question in this rapidly developing field is whether patients should be recontacted by professionals when there are new developments or when new (genetic) information becomes available, even after the initial consultation round up [6, 7]. Different types of information may trigger recontact: (1) new individual test results such as a reclassification of a genetic variant; (2) new information or treatment recommendations on a previous genetic result; (3) a new testing option such as a new discovered cancer gene [7–15]. There are strong arguments to support a prima facie moral duty to recontact, such as beneficence and respect for autonomy [16]. Also, empirical studies with different stakeholders such as healthcare professionals, researchers and patients show support for recontact [7, 17–24]. It is, however, also recognized that such a duty is complex [25], and in need of further interpretation, for it could otherwise be unlimited and overly demanding [7]. Earlier, we identified six contextual factors in the literature that may strengthen or weaken a duty to recontact: information features, costs and efforts, personal preferences, who is contacted, clinic or research setting, and time [16]. Although empirical studies on the viewpoints of different stakeholders show support for recontact [7, 17–24], further clarification is required about how stakeholders think about the extent and limits of recontact. Their opinions and moral intuitions about the reasons for and conditions under which to be recontacted are of importance for creating a realistic framework for clinical use. We therefore conducted focus groups with oncology patients and professionals in cancer care to explore their views on the contextual factors that may influence a duty to recontact in oncology care.

## 2. Materials and methods

### 2.1. Population

In order to collect viewpoints from different stakeholders, we invited oncology patients, former oncology patients and healthcare professionals to participate in our focus groups. The patient group was recruited from a health institute database consisting of patients and former patients, who agreed to be contacted about research projects. An email was sent with brief information about the focus group, after which patients could indicate whether they were interested in participating. Potential participants received detailed information about the focus group. For the two focus groups with professionals, we invited professionals from two different hospitals with extensive experience on oncological care. We aimed to encourage a conversation between people from different professional specialties since they were likely to put forward different viewpoints. Therefore, both groups with professionals were purposefully sampled in order to ensure a diverse professional background in each group. In all three focus groups only competent adults who spoke Dutch were included.

### 2.2. Focus groups/data collection

The focus groups were held at the corresponding institution, conducted in May and June 2019 and lasted 60–90 minutes. All participants provided their name, gender and age. Participants were given the opportunity to ask questions and subsequently signed informed consent forms. One researcher (NG and ALB) moderated the discussion; one researcher took notes and offered administrational support (NG and LSA). The focus groups were divided into three parts: (1) short introduction on recontact in genetics, (2) general thoughts of the participants on recontact (3) contextual factors that could influence a duty to recontact (see supplementary file 1). The focus groups were audio-recorded, transcribed verbatim, and stored coded.

### 2.3. Data analysis

The data analysis was based on the constant comparative method, which involves going back and forth from the data to develop codes and themes [26]. NG and LSA independently coded the full transcripts by labeling units of texts that referred to one or more topics relevant to the study purpose. Coding was done with QSR International’s NVivo 12 Pro version software. NG and LSA checked coded for consistency. After consensus on coding was reached, the codes were developed into higher-order concepts and themes. Subsequently, the themes for discussion were discussed with all authors.

## 3. Results

In total, three focus groups were conducted. One focus group consisted of 12 (former) oncology patients and one relative (Table 1). Two focus groups were conducted with professionals from two different hospitals: one group consisted of six professionals and one of seven professionals (Table 2). In all three focus groups, participants expressed that recontacting in (oncology) genetics is an almost incomprehensible and very complex topic (Table 3; Quote 1). At the same time, discussing recontact was considered important due to increasing amount of (genetic) information that becomes available.

**Table 1.** Participant characteristics: focus group with patients.

Participants	n = 12
Sex	
Male	7
Female	5
Age	48–71 yrs.
Patient / relative Patient	11
Patient relative	1

**Table 2.** Participant characteristics: two focus groups with professionals.

Participants	n = 13
Sex	
Male	3
Female	10
Age	39–59 yrs.
Professional background	
Surgeon	3
Oncologist/pulmonologist	2
Clinical geneticist	2
Nurse practitioner breast cancer care	2
Pathologist	1
Laboratory specialist clinical genetics	1
Ethicist	1
Social worker	1

**Table 3.** Illustrative quotes about recontacting, translated from Dutch.

Quote 1	“I find the topic quite difficult, because there are so many different situations imaginable. It is actually quite hard to comprehend. All the information that could potentially be discovered, and, uhm, I find it difficult to discuss this topic in general terms. However, I do realize that these issues exist, cause the possibilities expand.” Professional
Quote 2	“What would be the required benefit before you recontact someone, what is the minimum before you call someone?” Professional
Quote 3	“But I think that if you find something that is very important, such as a BRCA-mutation that was a class 3 and now a class 4, or a BRCA-mutation that was not found at first, and now is discovered for whatever reason, and it is really found in the patient, then you have to call that patient.” Professional
Quote 4	“A new insight to a former variant must be discussed. But if it concerns new testing options, well, then you could never stop. I mean then we would have to… every two years 20.000 people….” Professional
Quote 5	“That they would say there is a database, complemented with all sorts of developments concerning your illness, you could always check yourself. Everyone has access to that information. Then you wouldn’t have to approach everyone.” Patient
Quote 6	“You could always ask. Do you want to know? I, actually, always want to decide for myself if it affects my life.” Patient
Quote 7	“I think that If it only concerns yourself, you should be able beforehand to state whether you want that type of information or not. As soon as it concerns matters with a heritability trait, I think that a doctor, or the one who has the information, the professional, should always tell the person, for the sake of future generations.” Patient
Quote 8	“In practice, it is not always easy for patients to be offered that many options.” Professional
Quote 9	“Yes, so the wish is one time…, the wish is expressed one time, that doesn’t mean that it would persist indefinitely.” Professional
Quote 10	“You consent, based on the present-day information. If the world would change, well, then you could not assume that the consent applies to a totally different situation.” Professional
Quote 11	“What I like is that, well, … if the tumor is tested on genetic level, then we have to ask people, if they participate in the study, if they want to be informed, to what extent, if there are new results in the future. That indicates in a way the degree in which a person wishes to be informed.” Professional
Quote 12	“So actually I think, with these type of matters, actually, you could just not recontact patients, because you just don’t know if they want the information you will give them.” Professional
Quote 13	“But let’s discuss a sad example. The day will come that my wife and I will not be there anymore. And after it is determined that there is something hereditary. Then I would like for my daughter to hear about it. The patient is not present anymore, for whatever reason, out of the picture, then I would like that the information gets where it needs to be. So, I think it’s too limited to only focus on the doctor-patient.” Patient

In general, there was support to contact patients with certain (genetic) oncological information. The scope and extent of this duty to recontact was perceived differently. Some argued that recontact is only appropriate in extreme examples, e.g., certain lifesaving information, while others were inclined to approach former patients sooner. In the content analysis, no additional contextual factors were deduced from the focus groups that may affect the need to recontact patients. Hence, below the reflections of the focus group members on the six formerly mentioned contextual factors (textbox 1) are discussed in more depth.

### 3.1. Information features

The patient group made a distinction between new information that could be acted upon and information without obvious actionable consequences. There was agreement that they want to hear information that leads to screening or treatment options for themselves or their relatives. By contrast, not all patients want to hear new information in case there are no screening or treatment options. But it was stressed that preferably it would be up to themselves to decide whether they are informed about this type of information or not.

In the groups with professionals the relevance of new information was considered of great importance. The relevance was linked to the probability of a health risk and the possibility to act upon it. It was discussed that there are different levels of relevancy. In both groups this was illustrated by comparing different genetic oncology traits with different health risks, e.g., mutation in one of the BRCA-genes or in the CHEK2-gene. There was especially discussion on whether it would be possible to determine a certain threshold of expected health gain before a patient was to be recontacted (Table 3; Quote 2). In addition, some professionals put forward that the new information should be in line with the initial goal of the test. For instance, if a patient received a genetic test aimed at hereditary breast cancer, only information related to breast cancer should be reported. Furthermore, some stated that it is especially important to recontact a patient when the new information is a revision of a former result, e.g., reclassifying a variant in a BRCA-gene from class three to class four (Table 3; Quote 3).

### 3.2. Costs and efforts

In the patient group a tension between financial constraints and providing the best possible healthcare arose. Regarding the limited budget, it was recognized that costs and efforts play a crucial role in recontacting patients. However, it was particularly stressed that improving people’s health is the key goal of medical care. Since informing people about important health knowledge is part of that goal, this justifies certain efforts and costs. Moreover, it was emphasized that there are financial benefits for society when people know they have a high(er) risk to develop cancer and can take preventative measures.

In the groups with professionals, it was also discussed that there must be a balance between the costs and efforts to recontact people versus the expected benefits. In the discussion the limited means to recontact patients was more accentuated compared to the patient group. Furthermore, it was stressed that in practice it is not feasible for professionals to inform all persons about all new developments all the time (Table 3; Quote 4). Lastly, participants from all groups, came up with ideas to decrease the costs and efforts, e.g., by building a database for patients to search for relevant developments on their own initiative (Table 3; Quote 5).

### 3.3. Personal preferences

In the patient group it was recognized that the needs and desires about future information can differ considerably between persons. Ideally this should be taken into account. They articulated a desire to at least be informed at the time of the initial testing that they can be contacted in the future. Some patients want to be asked about their preferences on future contact (Table 3, Quote 6). An exception was made by some participants, for information that is relevant for the health of their relatives: in that case, they thought the new information should be communicated even if this would be against their own wishes (Table 3, Quote 7).

In the groups with professionals there was agreement that the personal preference of patients to be recontacted or not, is essential. Contacting people who do not want to be contacted was considered harmful. There was, however, discussion about the value of asking patients about their recontact preferences at the time of initial testing. Several concerns were discussed. First, there are general concerns that offering people too much information and options at the time of initial testing, can create difficulties in itself (Table 3, Quote 8). Second, it was stated that personal preferences on recontact may change over time (Table 3, Quote 9). Third, it is impossible to predict what future discoveries will be and therefore people may not be able to make a truly informed decision about their preferences (Table 3, Quote 10). Brushing some of the objections aside, certain professionals suggested that asking patients about their recontact preferences is not about specifying and stipulating every possible future option but should be aimed at understanding what a person’s stance is on being recontacted (Table 3, Quote 11). Nevertheless, for certain professionals, asking informed consent for recontacting is not a viable solution and they thought the risk of recontacting people against their wishes is too high. For them this was an overriding argument against recontact altogether, besides exceptional lifesaving cases (Table 3, Quote 12). Interestingly, in the patient group there was much less worry about the harmful effects of recontacting people against their wishes.

### 3.4. Who is contacted: the patient or a family member?

Particularly genetic information can be relevant for family members. However, in oncological care, it is conceivable that the patient cannot be contacted since he or she passed away before the new information became available. The patient group was clear that if the new information can lead to health benefits for relatives, their relatives must be made aware (Table 3, Quote 13). In general, the professionals were quite hesitant on contacting family members directly. Especially since relatives did not have the opportunity to make their wishes known on receiving information (Table 4, Quote 1). At the same time, some mentioned that genetic testing is sometimes primarily done by patients to inform their relatives about possible health risks. Hence, for certain professionals, there are situations conceivable in which family members are directly approached, such as when a BRCA mutation is detected.

**Table 4.** Illustrative quotes about recontacting, translated from Dutch.

Quote 1	“I don’t think actively toward family members. Because the family did not provided consent.” Professional
Quote 2	“I believe that if they find something scientifically relevant, significant, if there are results from scientific research everyone should know. That could be by newspapers or television, and preferably the people whom it concerns from a hospital database a bit sooner.” Patient
Quote 3	“A doctor has a care relationship, patient. That is not the case for a researcher. So there is a different relation.” Professional
Quote 4	“Surely, it cannot be that on Monday you find something. As a doctor, you perform tests, and on Tuesday you do the tests with the same results, and then you would think, no, now I don’t.” Professional
Quote 5	“I consider it to be relevant always, no matter how long ago.” Patient
Quote 6	“In a way you will always perceive yourself as a patient.” Patient
Quote 7	“Well I guess it depends on when there was contact, I think that if it was two years ago, it would be a completely different story then when it was twenty years ago.” Professional
Quote 8	“The more time passed by, the more the patient provided consent based on old information instead of the new circumstances.” Professionals
Quote 9	“I suppose, that, from the idea that I consider this is such a complex issue, that it may be appropriate to limit it somehow. There are many examples of matters in our society that come with limits and that is a good thing I suppose.” Professional
Quote 10	“That you would say for CHEK2 ten years, and for ATM fifteen years.” Professional

### 3.5. Clinic or research setting

New information can be gained in a clinical context, but also in a research context. In the patient group there was consensus that it was irrelevant whether new information was acquired in a research or clinical setting: when there is new important health information, they should receive the information (Table 4, Quote 2). In one focus group with professionals, it was articulated that a duty to recontact is much stronger for doctors than researchers, based on the care relationship of physicians (Table 4, Quote 3). However, in the other group it was stressed that the boundary between clinic and research is blurred (Table 4, Quote 4).

### 3.6. Time

For patients, the length of time between the initial genetic test and the new information is trivial: if the information is relevant, it should be discussed (Table 4, Quote 5). Some patients put forward that a cancer diagnosis changes a person in a way he or she will always feel like a patient (Table 4, Quote 6). In the focus groups with professionals, some also mentioned that time is not an overriding factor to refrain from renewing contact. Others, however, stressed that the amount of time between the test and the new information did actually affect the recontact situation (Table 4, Quote 7). The impact of time was linked to the validity of a patient’s informed consent: the more time passed by, the more doubt about the legitimacy of a person’s prior consent (Table 4, Quote 8). Moreover, several professionals explored the idea of linking the degree of health benefit to the time limit for recontacting someone (Table 4, Quote 9). Furthermore, in one group of professionals it was discussed that there is value in closure of certain life phases, hence, it may be valuable to introduce a time limit after which a patient will not be recontacted anymore (Table 4, Quote 10).

## 4. Discussion

From the focus groups, differences and similarities in the interpretation of the six contextual factors between (former) oncology patients and professionals could be discerned.

In the patient group there was consensus that if information was clinically relevant, they should be able to receive this information, which is in line with other empirical studies [24, 27–30]. Also, there was emphasis on the importance of informing patients at the time of initial testing that they can be recontacted in the future. And some even articulated a preference to be offered a choice whether to be recontacted or not, which is also noted in other studies [21]. Hence, the two factors *information features* and *personal preferences* were considered important by patients when weighing the strength of a duty to recontact. In the groups with professionals, the factors *information features* and *personal preferences* were also highly valued. There was, however, more discussion on what the threshold of actionability should be on how to determine this, which is also mentioned by professionals in other studies [17, 31]. Furthermore, there were worries on how to take personal preferences of patients into account. The possibility of over-reliance on informed consent was articulated, especially since there can be years between consent and the new information, as is echoed as well by professionals in another empirical study [32]. Furthermore, professionals had concerns about contacting patients against their wishes and harming them with information they do not want to receive. Interestingly, patients seemed less worried about being harmed, as long as they were informed about the option of recontact at the time of initial testing, which corresponds with other studies [27, 33, 34].

In the patient group, it was recognized that (financial) means are limited in health care. However, the factor *costs and efforts* was not extensively discussed. In the professional groups there was more emphasize on striking a balance between expected health benefits and costs.

Furthermore, in the patient group, genetic information on cancer risks was considered of particular importance for relatives, and therefore the factor *who is contacted* was not considered an overriding factor to refrain recontact: if the information is important for relatives, they should also have access. This corresponds with an empirical study, in which relatives of deceased oncology patients are contacted about a BRCA2 mutation that is discovered in the DNA of the deceased patient[35]. Also, a study with patients previously counseled about colon cancer risk, showed that new information on cancer risks for relatives is a specific reason why patients want to be recontacted [27]. For some professionals the factor *who is contacted* was an overriding factor not to recontact, in the sense that contacting family members is a bridge too far. At the same time, some professionals recognized that genetic testing was often done by cancer patients particularly for their relatives. The threshold to contact them, may, however, need to be higher.

For patients, the factors *time* and *clinic or research* setting were considered irrelevant altogether. The factor *research or clinical context* played a more important role in the discussion amongst professionals compared to the patient group, as is also echoed in other empirical studies [32]. However, it was also stressed that the dichotomy between clinical care and research is not always that clear, which other empirical studies on recontact point out as well [36]. Furthermore, for professionals, *time* itself was not considered an overriding factor not to recontact. Nonetheless, it was argued that as more time went by between the initial test and the new information, this complicates recontact matters, possibly to a point that recontact would not be possible anymore.

The results of this study provide insight in how the earlier formulated six contextual factors [16] are interpreted by different stakeholders in oncology care. In sum, in the patient group there was a general tendency that clinically important information should be available to patients. The group attributed little meaning to the *nature* of the relationship between the professional who gained new information and the person who would be contacted. Also, without a clear care relationship, e.g., in research settings or when a relative would be contacted, the patient group regarded recontact for clinically important information appropriate. The group did, however, value being informed about the option of being recontacted in the future, or asked for their permission, at the time of initial testing. In the groups with professionals, there was also a general tendency that clinically important information should be shared with patients. There was, however, more doubt about which information requires recontact. In contrast with the patient group, the nature of the relationship between the professional and the person who would be contacted, was a subject of discussion, i.e., they put more emphasize on whether there was a care relationship. Furthermore, while also attributing significant importance on a person’s preference about being recontacted, there was worry about the value of informed consent at the time of initial testing.

Another lesson learned from this study is that oncology patients were quite clear in their wish to receive relevant updates and that the professionals were more hesitant to consider recontact as a standard of care, which was also found in other studies [22]. This is not surprising as recontacting patients with new information would mean a shift from a *patient-initiated* driven approach toward an *information*-*initiated* driven approach in health care. Instead of the patient initiating contact when he or she has a health-related question, the professional is the initiator of contact when new relevant information is available. This way of offering healthcare is more in line with the framework and goals of public health, but less with current clinical care. Since this would entail a different way of offering healthcare, it is doubtful whether the complete solution to the recontact debate lies within the attempt to demarcate a professional’s duty to recontact solely. If we would agree that patients should receive updates that are important to their health, this requires a profound change of offering healthcare. The question would not only be what the professionals’ responsibilities are in their current work forms, but how to design a system that complies with patients’ wishes (or perhaps rights) to receive updates. This development is also hinted upon by the participants themselves when they propose to build databases of digital solutions to decrease costs and efforts of recontacting. Before implementing such systems, it would be helpful to conduct feasibility studies to determine, among other factors, the workload of physicians, IT-possibilities and the skills of patients to understand and use these databases.

## 5. Conclusions

The earlier formulated six contextual factors can support a healthcare professional in weighing their responsibility to recontact [16]. The results of this study provide insight in how the factors are interpreted by different stakeholders in oncology care. Oncology professionals can make use of these results when confronted with new information. Moreover, the results can be used as input for the development of professional oncology guidelines on recontact. For example, to adapt to personal preferences, streamline processes to reduce physicians’ workload, to establish reasonable cutoff points for recontacting and determine when renewed consent of patients is desired. Furthermore, it is advisable to at least mention the possibility of recontacting at the time of initial genetic testing. Lastly, the study underscores that recontact is a different approach to healthcare than the traditional model in which a patient initiates contact. This paradigm shift requires further exploration through interviews and focus groups with various stakeholders (i.e., patient relatives and the general population), to assess its desirability and feasibility.

## Article highlights

### Introduction

- In genetics, former results can gain new meaning in the course of time. This raises questions about when professionals should recontact patients with new information.
- The aim of this focus group study is to clarify how different stakeholders in oncology think about the extent and limits of a duty to recontact.

### Materials and methods

- One focus group with oncology patients (n = 12) and two groups with healthcare professionals (total n = 13) were conducted.

### Results

- There are differences and similarities between patients and professionals on how they interpret the extent and limits of a duty to recontact based on the contextual factors: information features, costs and efforts, personal preferences, who is contacted, clinic or research setting, and time.
- Oncology patients were quite clear in their wish to receive updates while the professionals were more hesitant to consider recontact as a standard of care.

### Discussion

- Recontacting patients with new information would mean a shift from a *patient-initiated* approach toward an *information-initiated* approach. This entails a different way of offering healthcare.
- The question is not only what professionals’ responsibilities are, but how to design a system that complies with patients’ wishes to receive updates.

## Supplementary Material

Supplemental Material
