# Supplementary material for: DNA sequencing in oncology: a focus group study on a duty to recontact
Source: Future Sci OA. 2024 Nov 22;10(1):2432233. doi: 10.1080/20565623.2024.2432233 (PMC11587842; doi:10.1080/20565623.2024.2432233)
Supplement: Supplemental Material [file IFSO_A_2432233_SM4984.pptx]

## Slide 1
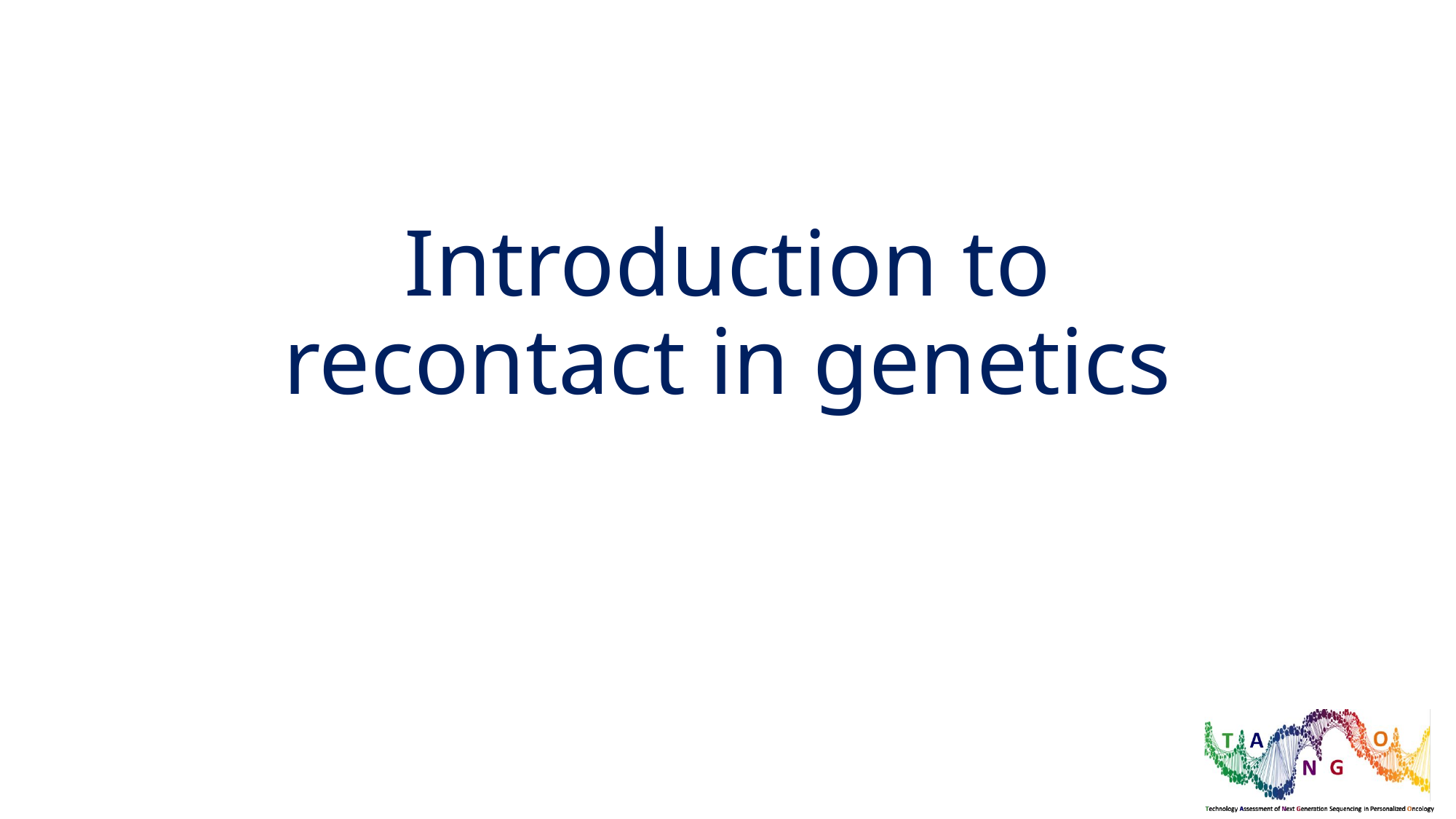

# Introduction to recontact in genetics

## Slide 2
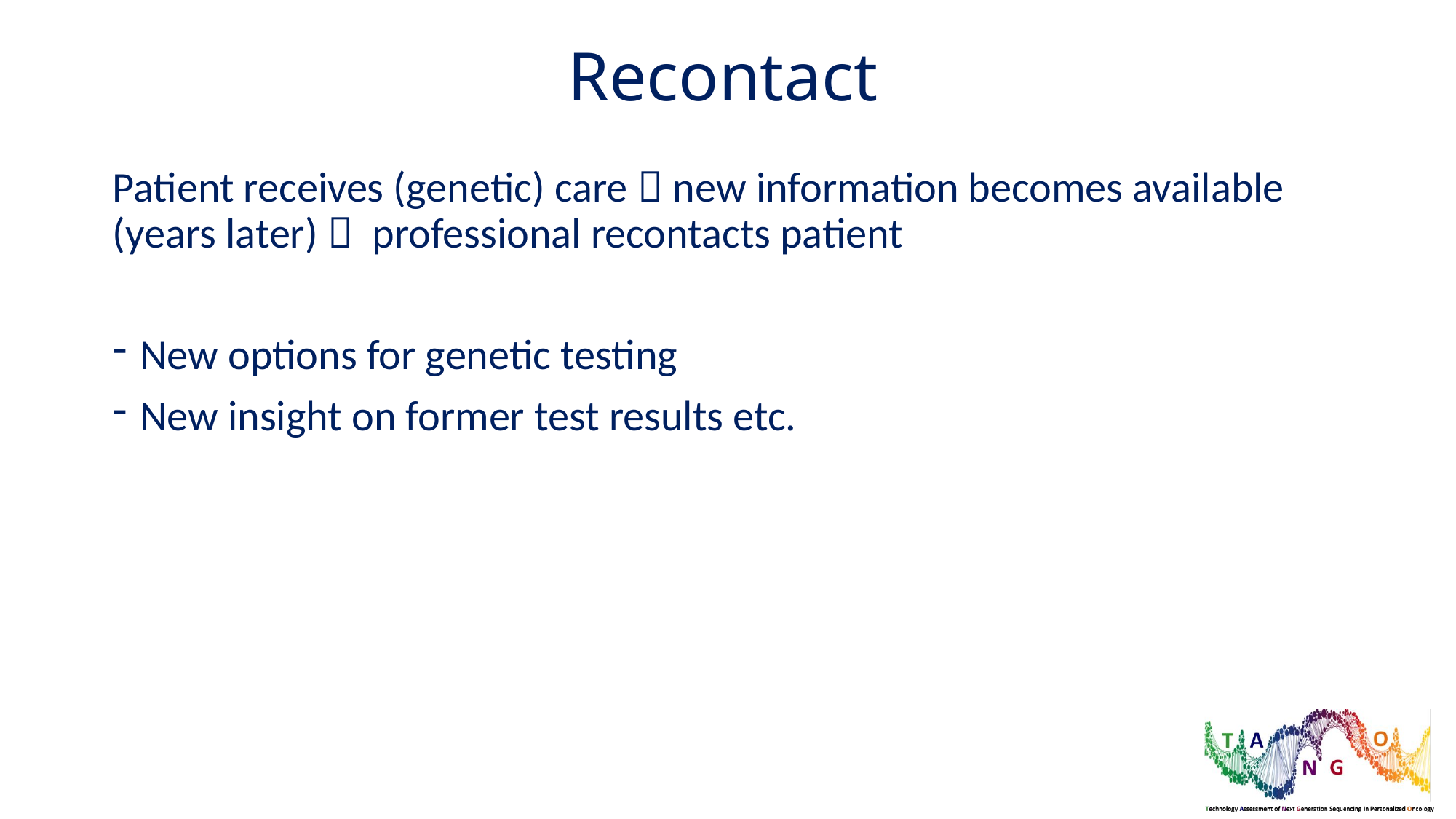

# Recontact
Patient receives (genetic) care  new information becomes available (years later)  professional recontacts patient
New options for genetic testing
New insight on former test results etc.

## Slide 3
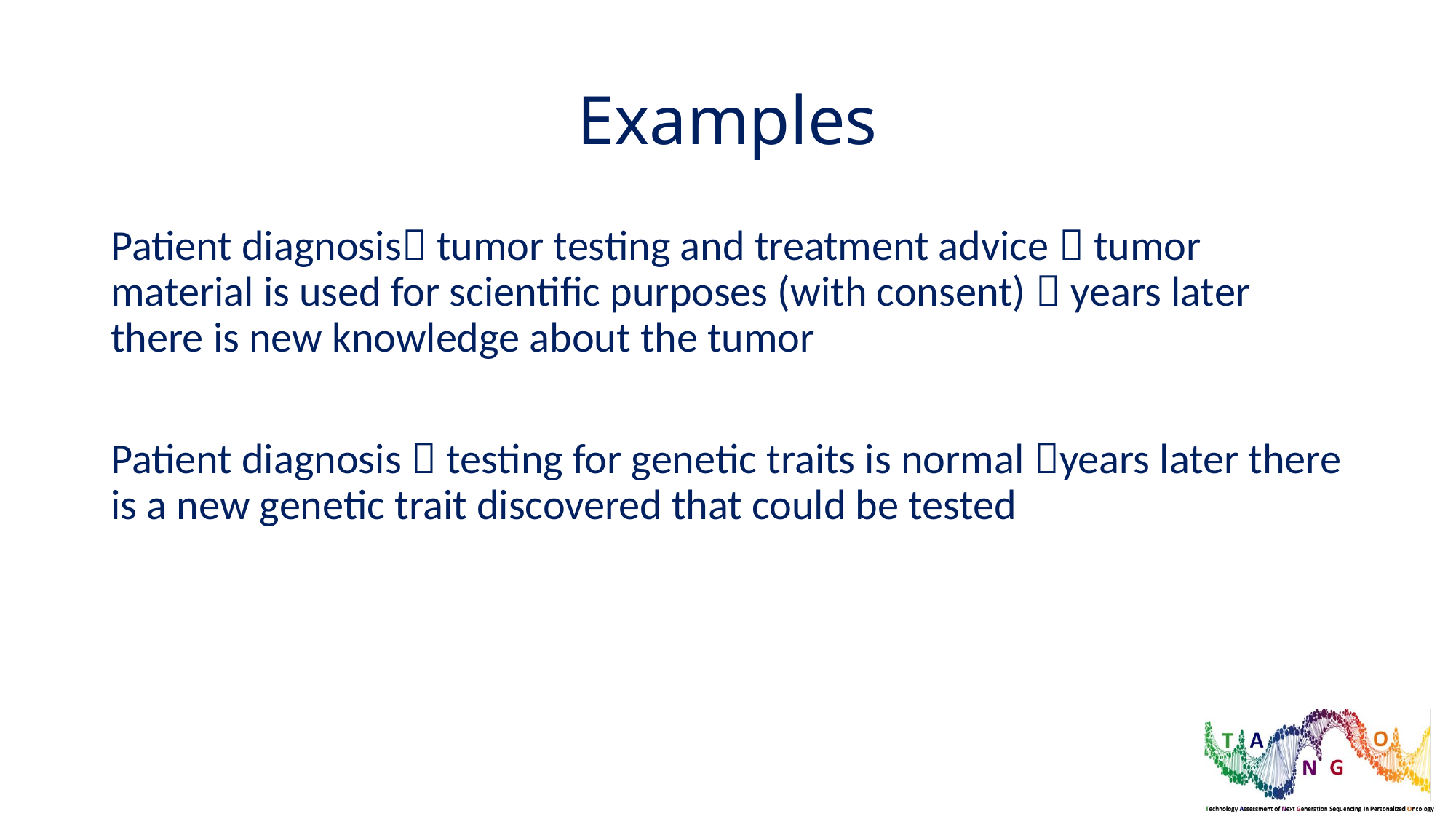

# Examples
Patient diagnosis tumor testing and treatment advice  tumor material is used for scientific purposes (with consent)  years later there is new knowledge about the tumor
Patient diagnosis  testing for genetic traits is normal years later there is a new genetic trait discovered that could be tested

## Slide 4
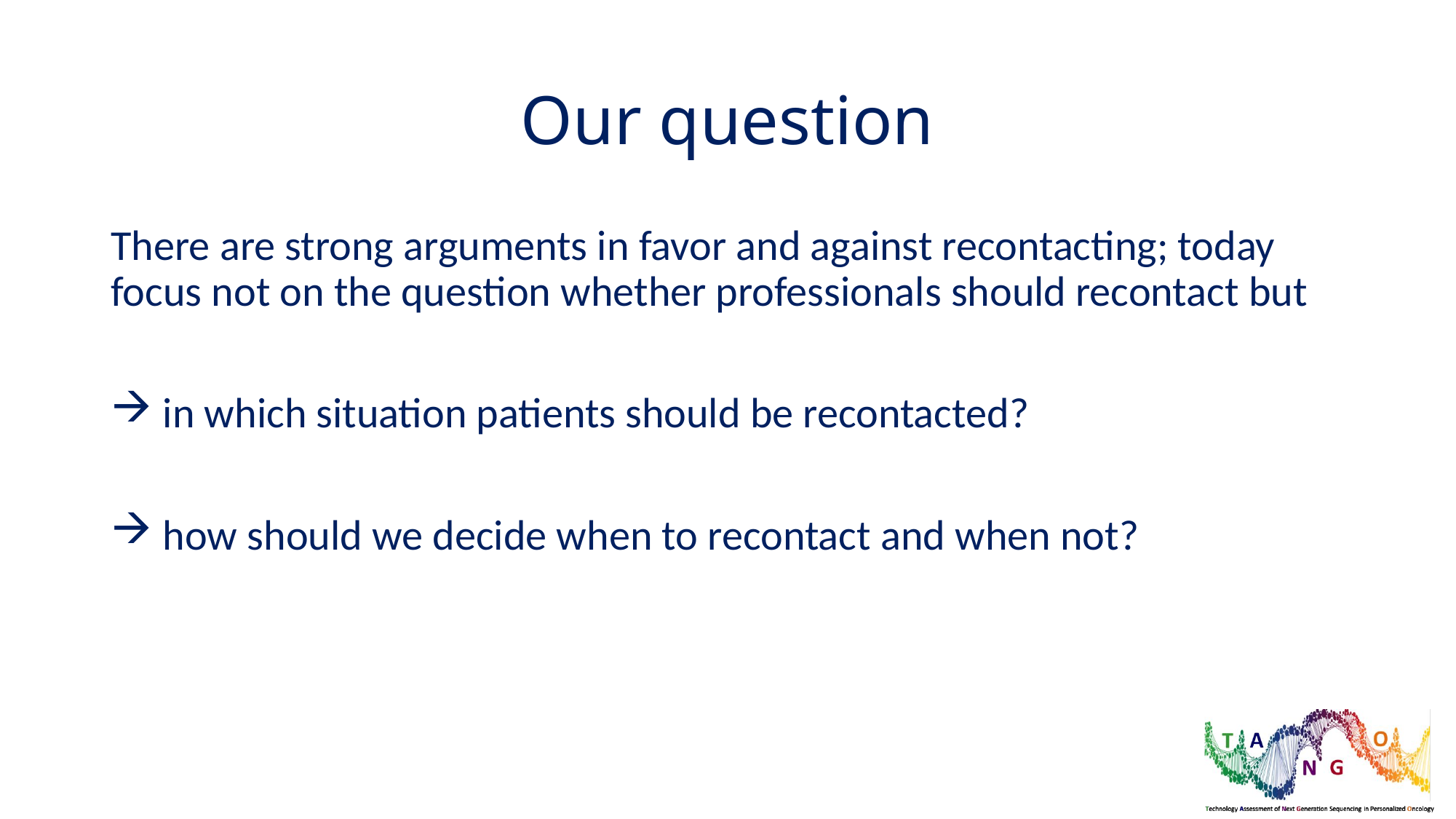

# Our question
There are strong arguments in favor and against recontacting; today focus not on the question whether professionals should recontact but
 in which situation patients should be recontacted?
 how should we decide when to recontact and when not?

## Slide 5
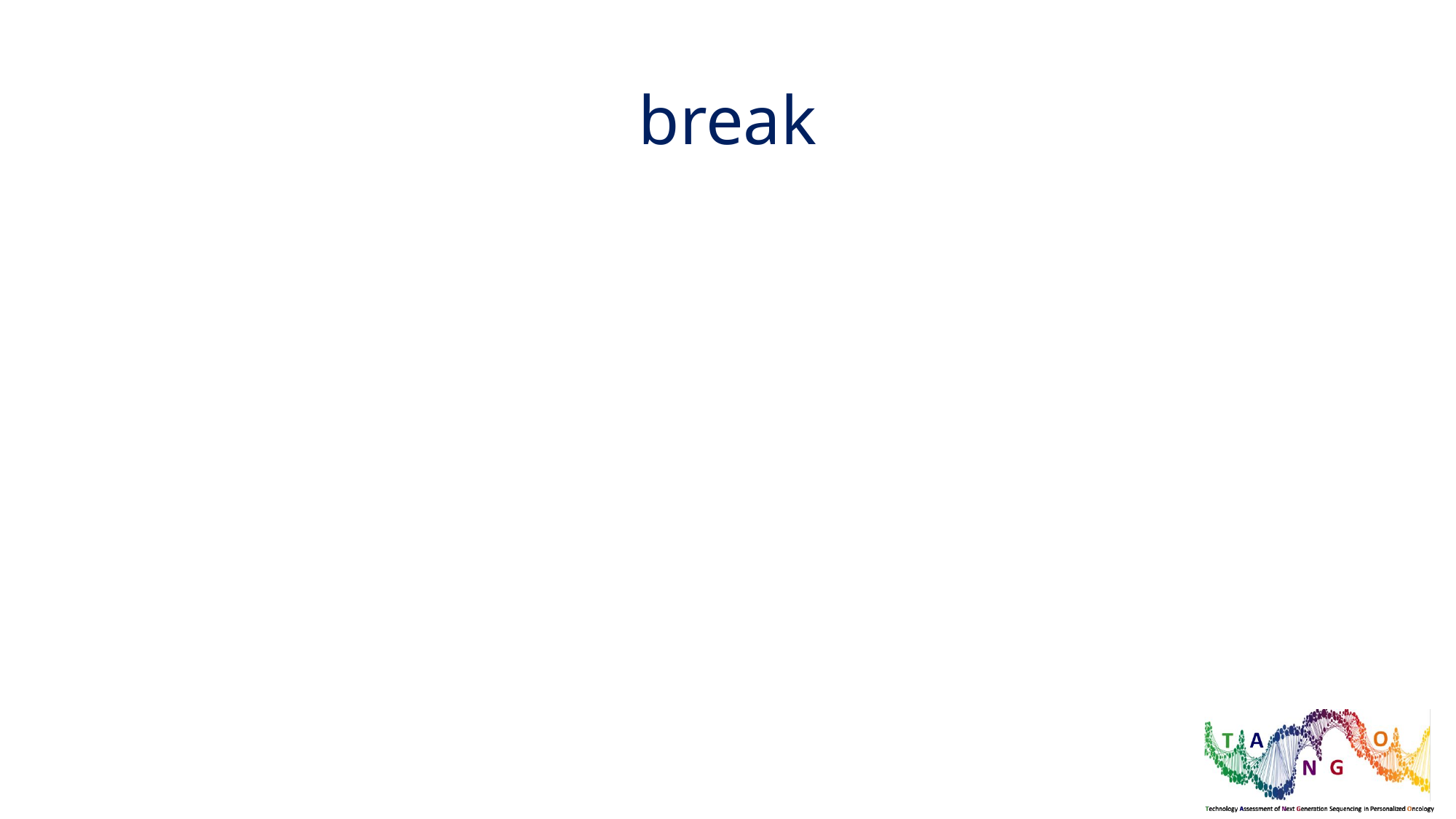

# break

## Slide 6
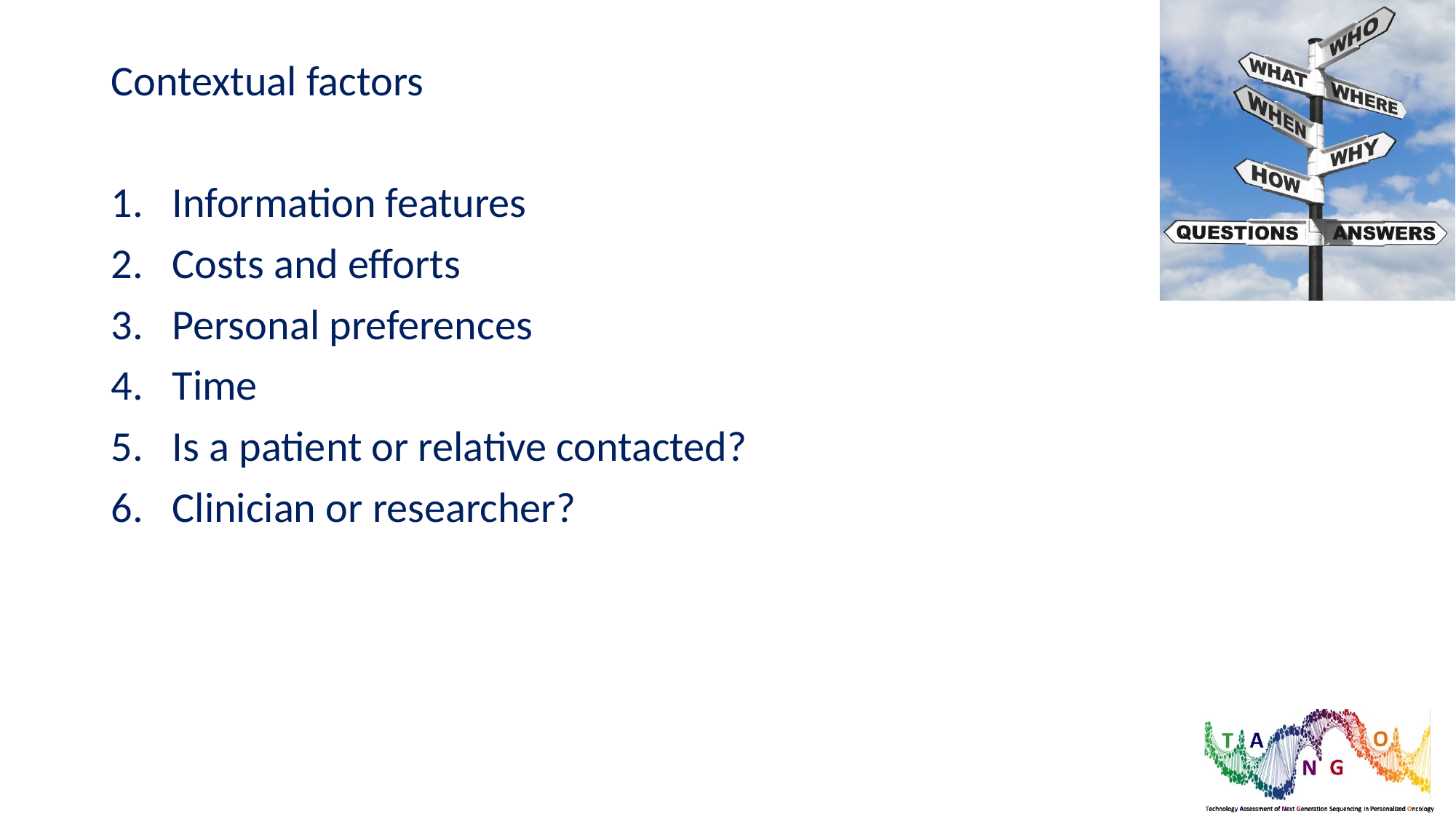

Contextual factors
Information features
Costs and efforts
Personal preferences
Time
Is a patient or relative contacted?
Clinician or researcher?

## Slide 7
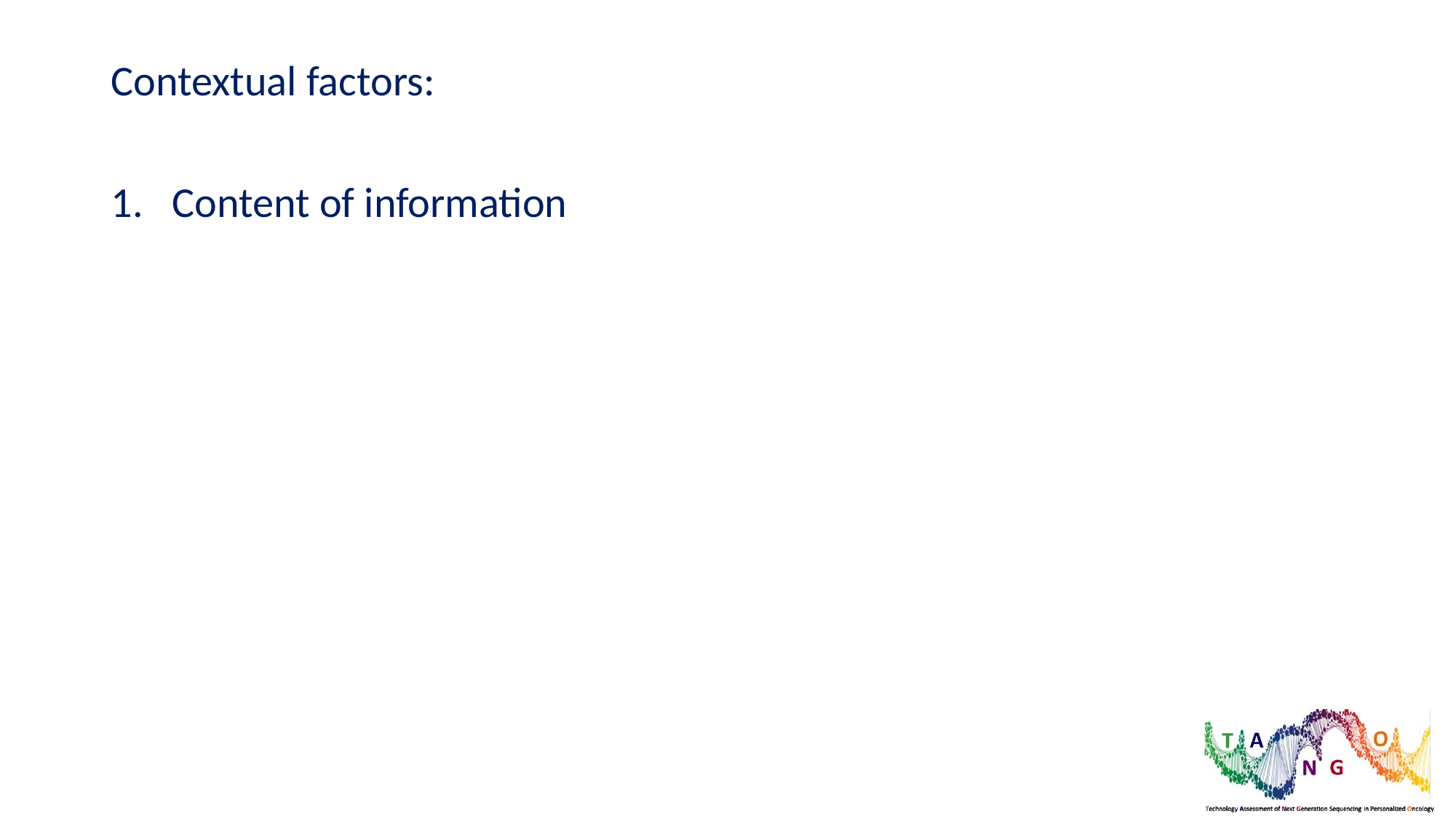

Contextual factors:
Content of information

## Slide 8
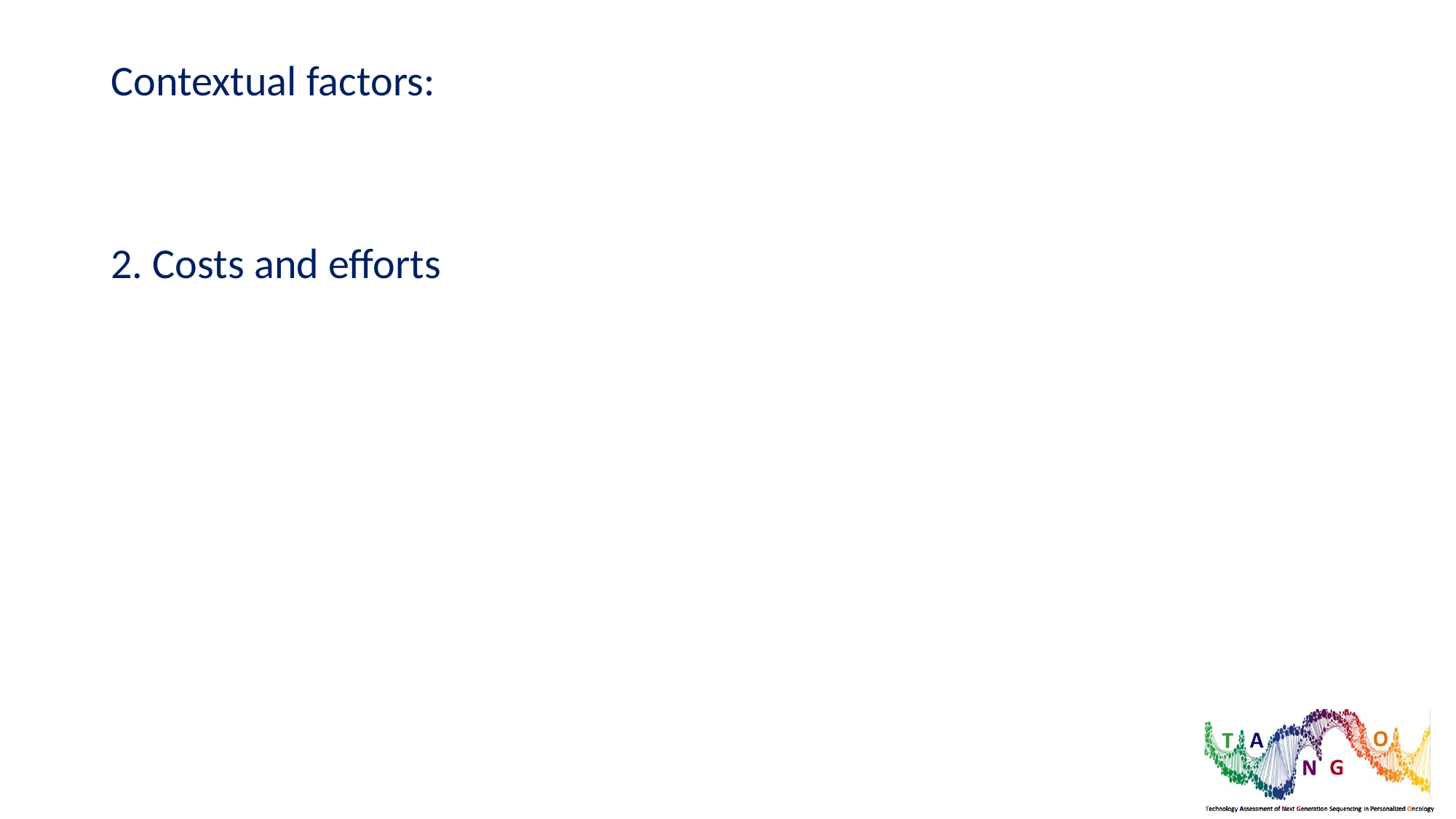

Contextual factors:
2. Costs and efforts

## Slide 9
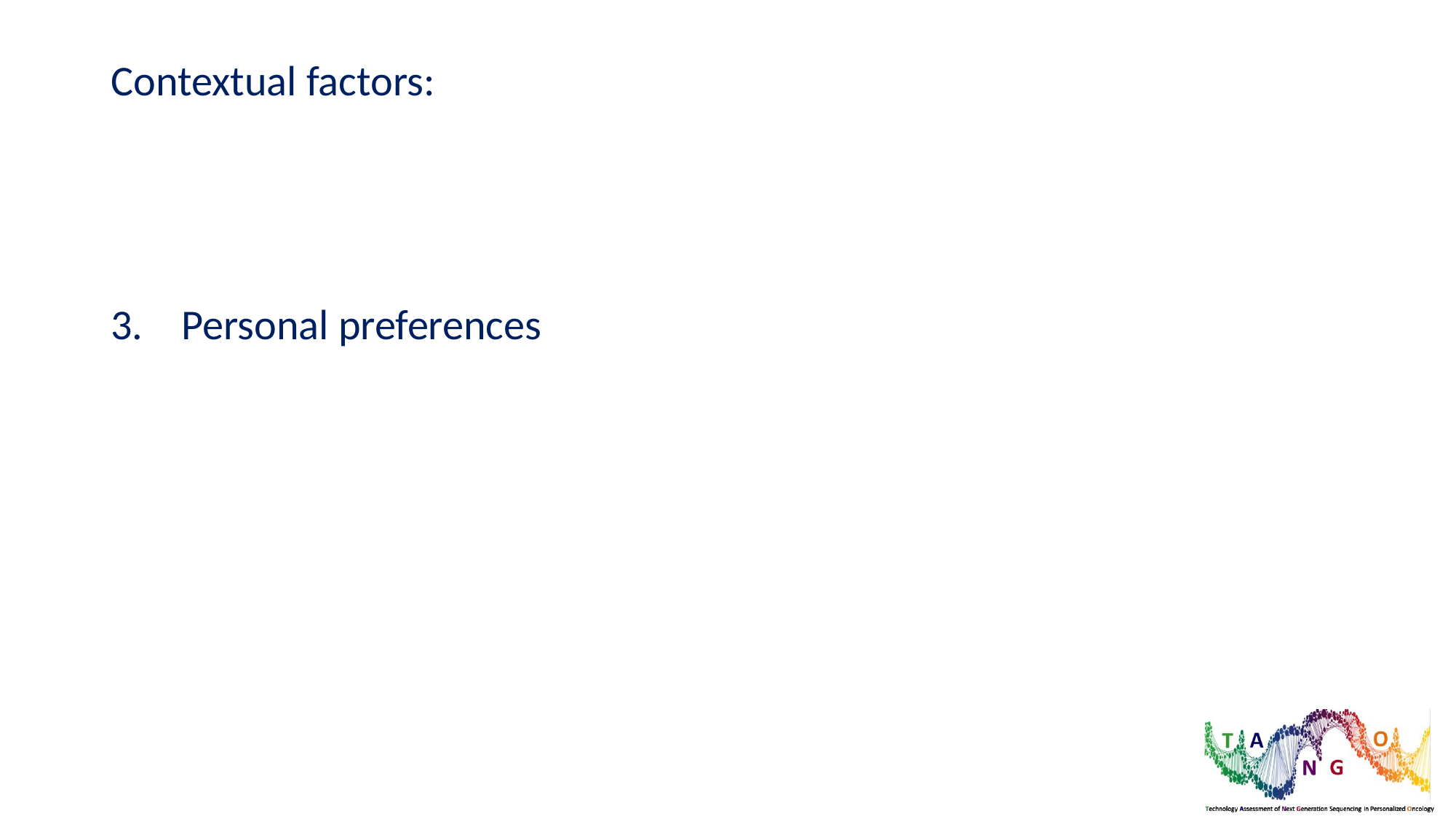

Contextual factors:
3. Personal preferences

## Slide 10
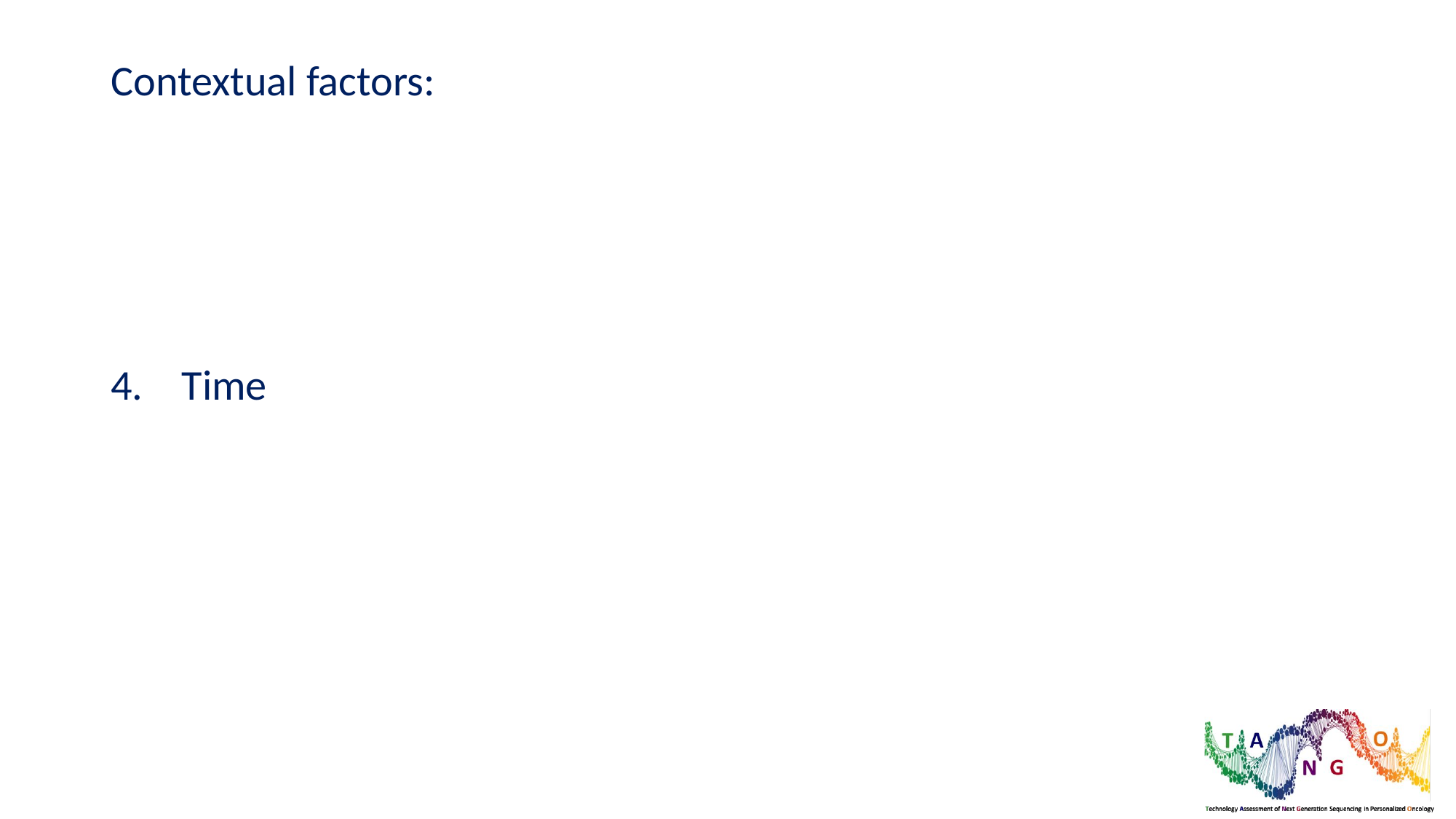

Contextual factors:
4. Time

## Slide 11
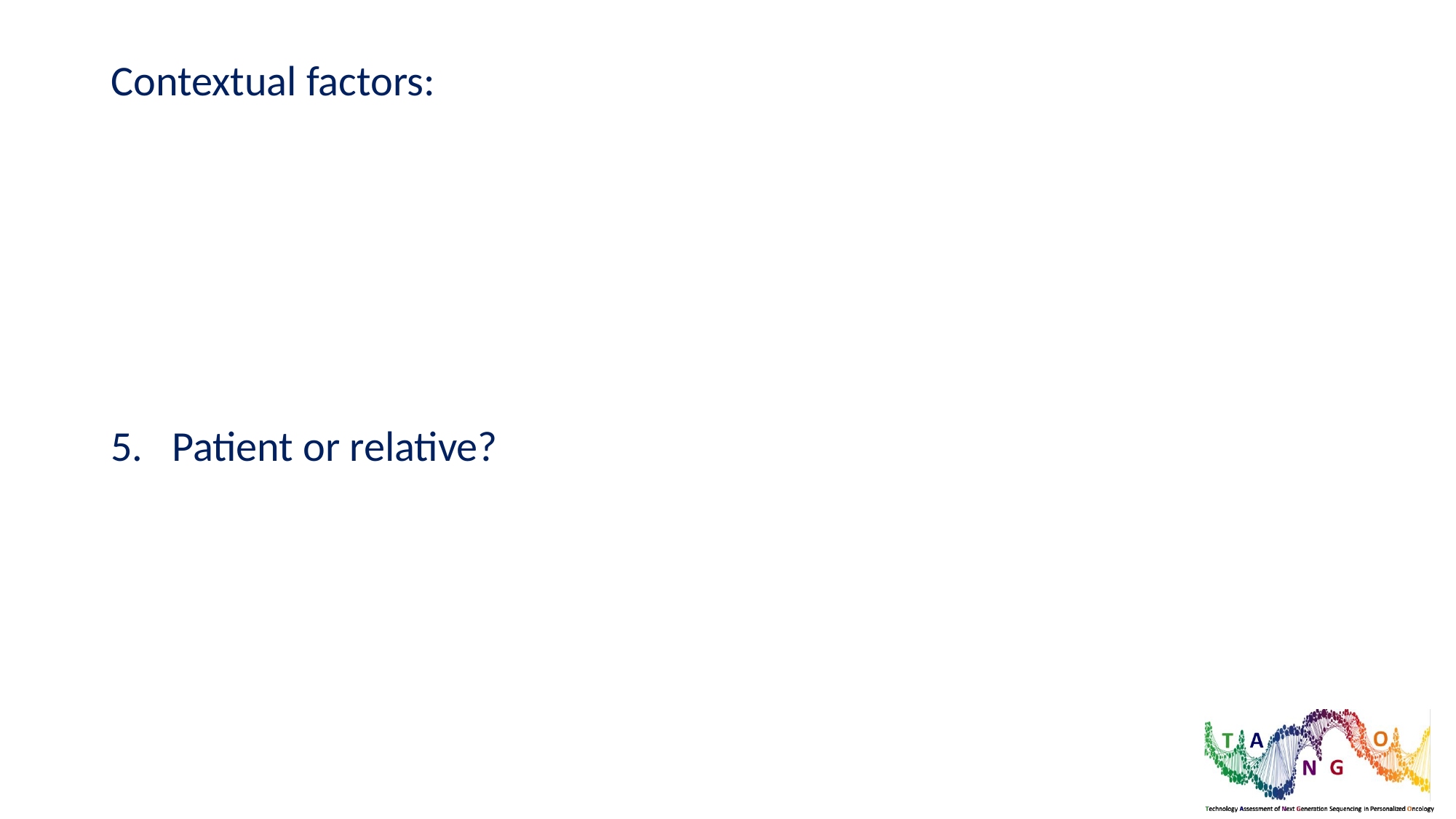

Contextual factors:
5. Patient or relative?

## Slide 12
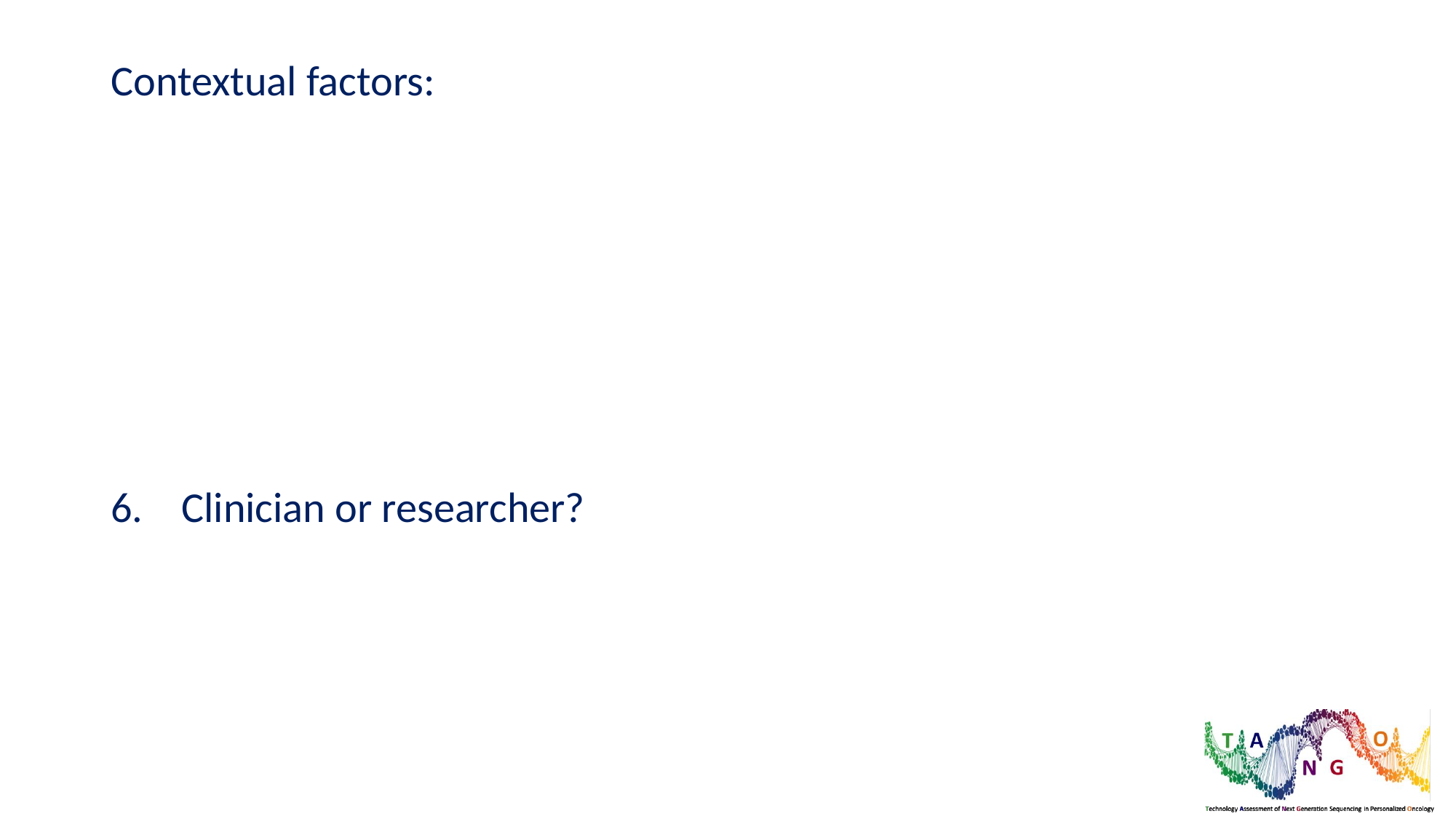

Contextual factors:
6. Clinician or researcher?

## Slide 13
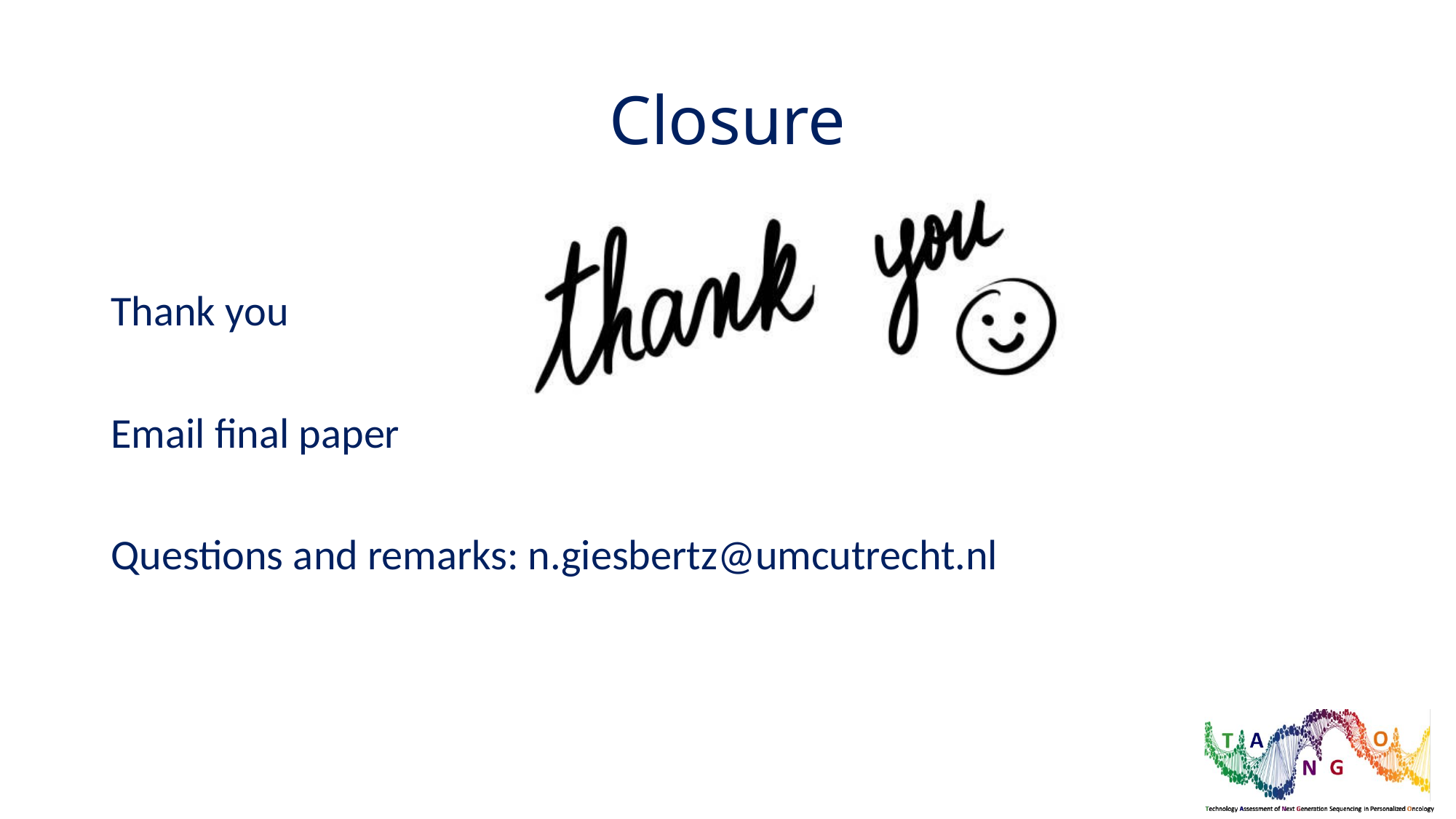

# Closure
Thank you
Email final paper
Questions and remarks: n.giesbertz@umcutrecht.nl
